# Supplementary material for: Content-rich biological network constructed by mining PubMed abstracts
Source: BMC Bioinformatics. 2004 Oct 8;5:147. doi: 10.1186/1471-2105-5-147 (PMC528731; doi:10.1186/1471-2105-5-147)
Supplement: Additional File 5 — The original Chilibot query results of the term "long-term potentiation (LTP)" and 22 other terms, limiting the latest references analyzed to the years 1990, 1995, 2000, and 2004. [file 1471-2105-5-147-S5.bz2 › chilibotAdditionalFile5/ltp2000/html/TRKB_AMPA.html]

 


 **TRKB** and **AMPA** 
  
Found 9 abstracts in PubMed,  **9 abstracts were retrieved and analyzed**.  


---

 Search Google  |
 PDF files only 
|  EDU domain only 

---

**Interactive relationship** (e.g. stimulation, inhibition, etc)

- Moreover, analysis of  **TrkB**  expression in culture revealed a close correlation between the percentage of nTS neurons in which BDNF inhibits  **AMPA**  currents and the percentage of neurons that exhibit  **TrkB**  immunoreactivity.  Ref: 10684891 J Neurosci, 2000
- Thus, the impaired  **AMPA**  receptor function in the stg mutant GC is not likely to result from the reduced BDNF  **TrkB**  signaling.  Ref: 10407040 J Neurosci, 1999

- :-)
